# Community suicide rates and related factors within a surveillance platform in Western Kenya

**DOI:** 10.1186/s12888-021-03649-6

**Published:** 2022-01-04

**Authors:** Linnet Ongeri, David A. Larsen, Rachel Jenkins, Andrea Shaw, Hannah Connolly, James Lyon, Symon Kariuki, Brenda Penninx, Charles R. Newton, Peter Sifuna, Bernhards Ogutu

**Affiliations:** 1 Kenya Medical Research Institute, Centre for Clinical Research, Nairobi, Kenya; 2 Syracuse University Department of Public Health, Syracuse, NY USA; 3 Kings College London, London, UK; 4 Institute for Global Health and Translational Science, SUNY Upstate Medical University, Syracuse, NY USA; 5 Kenya Medical Research Institute, Wellcome Trust Program, Kilifi, Kenya; 6 Vrije University, Amsterdam, Netherlands; 7 US Army Medical Research Directorate–Kenya (USAMRD-K)/Kenya Medical Research Institute (KEMRI), Kisumu, Kenya

**Keywords:** suicide, Health Demographic Survey System, Kenya, risk factors, mental disorders, verbal autopsy

## Abstract

**Background:**

Suicide is an important contributor to the burden of mental health disorders, but community-based suicide data are scarce in many low- and middle-income countries (LMIC) including Kenya. Available data on suicide underestimates the true burden due to underreporting related to stigma and legal restrictions, and under-representation of those not utilizing health facilities.

**Methods:**

We estimated the cumulative incidence of suicide via verbal autopsies from the Health and Demographic Surveillance System (HDSS) in Kisumu County, Kenya. We then used content analysis of open history forms among deaths coded as accidents to identify those who likely died by suicide but were not coded as suicide deaths. We finally conducted a case-control study of suicides (both verbal autopsy confirmed and likely suicides) compared to accident-caused deaths to assess factors associated with suicide in this HDSS.

**Results:**

A total of 33 out of 4306 verbal autopsies confirmed suicide as the cause of death. Content analysis of a further 228 deaths originally attributed to accidents identified 39 additional likely suicides. The best estimate of suicide-specific mortality rate was 14.7 per 100,000 population per year (credibility window = 11.3 – 18.0). The most common reported method of death was self-poisoning (54%). From the case-control study interpersonal difficulties and stressful life events were associated with increased odds of suicide in both confirmed suicides and confirmed combined with suspected suicides. Other pertinent factors such as age and being male differed depending upon which outcome was used.

**Conclusion:**

Suicide is common in this area, and interventions are needed to address drivers. The twofold increase in the suicide-specific mortality rate following incorporation of misattributed suicide deaths exemplify underreporting and misclassification of suicide cases at community level. Further, verbal autopsies may underreport suicide specifically among older and female populations.

## Background

Suicide is one of the leading causes of death worldwide and the fourth leading cause of death for adolescents and young adults aged 15-19 years [1] . Globally, an estimated 700,000 persons die from suicide related deaths annually, representing an annual global mortality rate of approximately 9.0 per 100,000 populations with majority (77%) of these deaths in low- and middle-income countries (LMIC) [2]. It is estimated that the African region carries the highest age standardized suicide rate globally (11.2 per 100,000), Europe (10.5 per 100,000) and S. E Asia (10.2 per 100,000) [2] . In the Eastern sub-Saharan Africa region, the age standardized mortality rate from suicide in 2016 was 12.5 per 100,000 populations for both sexes, higher in males at 18.7 per 100,000 compared to females at 7.0 per 100,000 populations [3]. Kenya’s average crude national suicide death rate is estimated at 6.1 per 100,000 populations with a male to female ratio of 3:1 [2]. Like many African countries, suicide data are scarce [4] and most estimates are based on audits from hospitals, hence may not be representative of the situation in the general population [5, 6]. Data on suicide rates when available, frequently underestimate the true occurence of suicide in a population [7–9]. Reasons for this include challenges in suicide reporting and societal attitudes to suicide [4, 7] . Suicide and suicidal attempt are not only highly stigmatized in Kenya but also illegal resulting in underreporting of cases in the vital registration systems [10]. Additionally, data on intent is not routinely captured in medical certification of death resulting in high misclassification of suicide cases.

Various factors have been documented to increase the risk of suicide in sub-Saharan Africa. Risk factors prominently outlined include alcohol and drug use [11–14], interpersonal and social difficulties [15, 16] as well as factors related to socio economic status [12, 14]. However, these factors are not fully understood in many poor settings and the factors may vary by culture and geographical location, hence prompting a need for context specific risk data [4]. Suicide is an important contributor to preventable mortality globally. However, the dearth of research reporting on suicide rates and its risk factors in the region and country can limit the evidence base for targeted and successful suicide prevention interventions. There is a critical need to understand the true burden of this public health problem to inform suicide prevention efforts. Verbal autopsy reports may provide a more accurate and detailed report on suicide related cause of death in Kenya.

In this paper we use mortality data from a Health Demographic Survey System (HDSS) in rural Western Kenya conducted using verbal autopsies to specifically report on incidence of suicide and associated risk factors over a 5-year period. We postulate that deeper review of content analysis of accidental deaths and deaths by “external causes” may further our understanding of the true burden of suicide and associated risk factors.

## Methods

### Study design

In this study a review of the verbal autopsy data or records from an HDSS cohort between 2011-2017 was conducted to estimate the cumulative incidence of suicide-specific deaths in the community. We first identified deaths labeled as suicide from verbal autopsies. We then conducted content analysis of verbal autopsy open narratives of deaths due to External Causes” (including accidents, injuries, assaults, and unspecified causes) to identify suspected suicides that were misclassified by verbal autopsy software. This approach allowed inclusion of deaths by suicide often missed by routine verbal autopsy categories. We subsequently conducted a case-control study of suicides compared to deaths from external causes to assess factors associated with suicide in this region.

### Setting

**Fig. 1:**
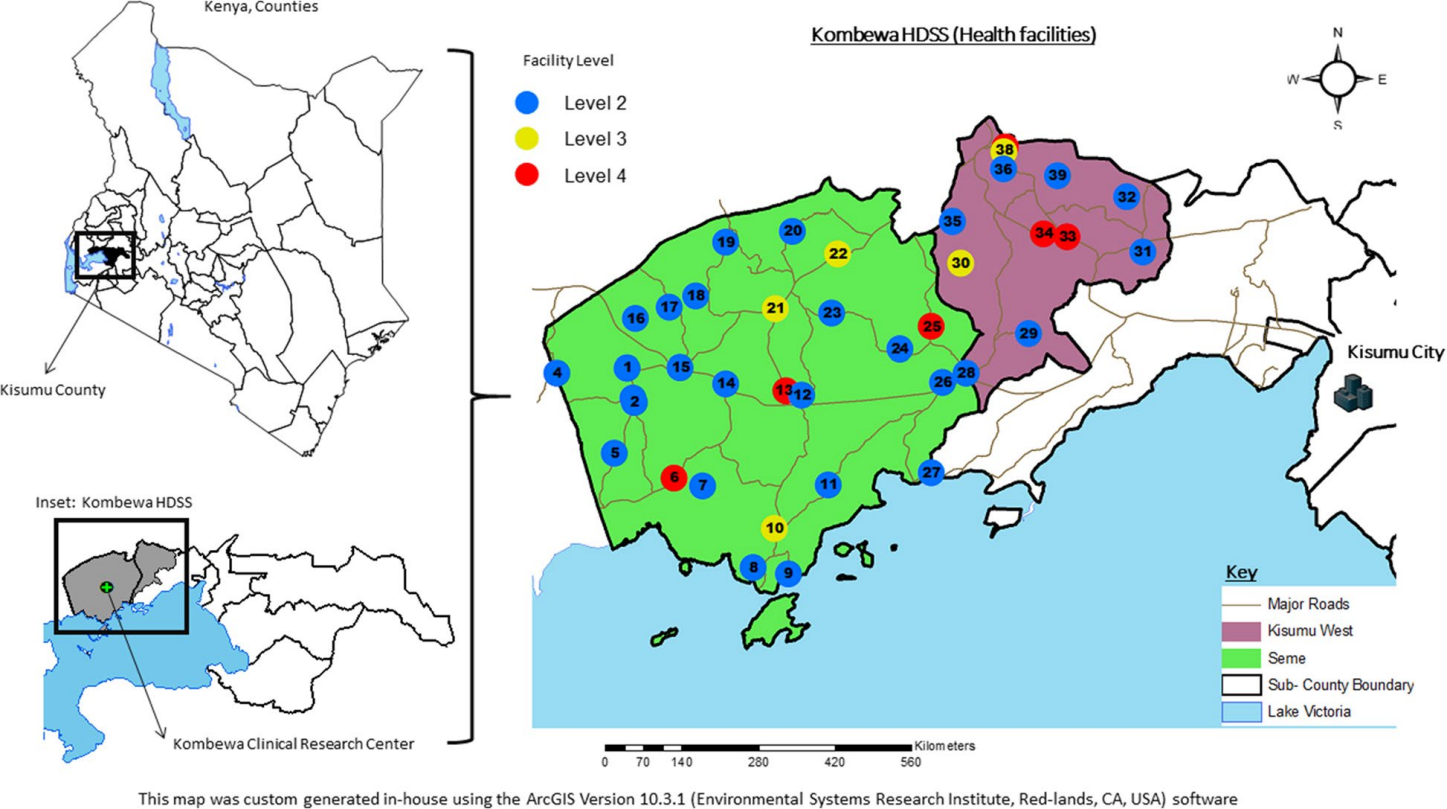
Map location of Kombewa HDSS

This study was conducted using data from the Kombewa HDSS run by the Kenya Medical Research Institute and Walter Reed Army Institute of Research (WRAIR) collaborative program, the United States Medical Research Directorate- Kenya (USMRD-K) in the rural Kisumu County, Western Kenya. Details of the Kombewa HDSS area have been described in detail elsewhere [17]. In brief, the Kombewa HDSS covers an area of about 369 km^2^ along the north-eastern shores of Lake Victoria (Fig. 1). Baseline data was collected in 2011 in about 40,000 households within the HDSS and afterwards they are visited every 6 months to document births, deaths, causes of death (verbal autopsy), health indicators, and various syndromes including fever, cough, and diarrhea. There are about 39 functioning health facilities within the HDSS, 30 of which are government and 9 private or faith-based organizations. The various health facilities are graded from level one to level four depending on the services offered. The private and faith-based health facilities complement the services offered by the government usually at a cost. The majority of them offer outpatient while a handful offer inpatient and laboratory services. Almost all facilities provide immunization services using the Expanded Programme on Immunization (EPI) vaccines. There are no specialized mental health services within the HDSS.

### Study Population

A dynamic cohort of 154,140 individuals (drawn from 38,275 households) in Seme and parts of Kisumu West Sub-Counties of Kisumu County are currently monitored in this prospective population-based surveillance platform. The population is primarily young, with a mean age of 23 years (48% of population below the age of 15). The area covered by the HDSS has an intense malaria transmission and high prevalence of Human Immunodeficiency Virus (HIV) infection [18, 19]. The HIV prevalence in the region encompassing the entire HDSS is three times the nationally reported average of 5.1% [20].

### Data sources / measurement

Residents in the HDSS area are defined as all persons residing in the study area for 6 months or more, excluding transient residents and visitors. In addition to the routine house visits, a team of dedicated "village reporters" provide birth and death notification within 7 days of the event. The notified events are thereafter verified and entered into the HDSS database by a team of dedicated staff. A notification of birth and death within 7 days of the event is provided by the village reporting team largely drawn from a pool of Ministry of Health trained community health workers. The deaths are verified and entered into the HDSS database and relatives of the deceased are then followed up with a standardized Verbal Autopsy (VA) interview by specially trained lay interviewers to record events surrounding death. VA was conducted using the modified 2007 and later 2012 standardized WHO questionnaires recommended by INDEPTH, for all deaths occurring in the HDSS [21, 22]. The VA tool consists of three separate VA questionnaires that are used to collect data on neonates (0-28 days old), children (29 days to 14 years old), and adolescents and adults (15+ years). The questionnaires contain a series of closed questions followed by an open history section. The closed questions elicit details of the illness and medical history, while the open history allows the respondent to describe the illness and events leading to the death from memory without structure. VA interviews are performed a few weeks after burial to respect the mourning period, while still facilitating recall. Absence of an adult in the home is recorded as “no respondent.”

Assignment of causes of death were made using the InterVA-4 model version 4.02. A detailed description of the InterVA model has been given elsewhere [22]. In brief, the InterVA-4 model is computer based, and uses the Bayes’ theorem, in an attempt to overcome the longstanding limitations of alternative methods [23–25], such as physicians coding. InterVA-4 ascertains probable cause(s) of death for each VA case, the workings being centered on a combination of expert medical opinion and relevant available data [26]. The causes of death generated by the InterVA-4 are compatible with the International Classification of Diseases version 10 (ICD-10) categorized into 62 groups as defined in the 2012 WHO VA instrument [21]. VA data obtained prior to the WHO 2012 standards at the site were retrospectively transformed into the WHO 2012 and InterVA-4 input format for processing.

Not all deaths in the HDSS over the study period received verbal autopsies. Due to funding constraints, HDSS activity was somewhat curtailed. If HDSS personnel were unable to investigate a death within one year, then no verbal autopsy was conducted to avoid recall error.

#### Identifying Likely Suicides Miscategorized by Verbal Autopsies

Upon review of all mortality reports recorded in the HDSS from 2011-2017, a subset of deaths was reviewed that included death by suicide, external cause (accidents, injuries, assaults, unspecified external cause) as well as those where verbal autopsy reporters answered “yes” to the question “Do you think the deceased committed suicide?”. From these deaths the details of the verbal autopsy data were reviewed, a detailed review of the medical history (including history of mental illness, depression, mental confusion), history of suicidal attempts or ideation, history of self-harm, respondents’ belief the person committed suicide, history of substance abuse, description of death in the open history form, were all taken into account [27, 28]. History of mental illness was presumed to include any mental illness that was fairly obvious to the informant. Two independent reviewers (J.L and H.C) then deemed these deaths to be a likely suicide or not. The two reviewers at the time of the review were medical students and had been trained by two psychiatrists (L.O and R.J) on an overview of suicidal behavior and factors associated, as well as contextualized phrases indicative of these factors. Percent agreement between the two reviewers stood at 96% and this was found to be satisfactory. In cases of discrepancy between independent reviewers, the case reports were discussed with a larger team and a conclusion reached on whether to categorize these as suspected suicides. Those that were not originally labeled by the verbal autopsy as suicide but suspected to be suicide by qualitative analysis were re-labeled as suspected cases. We classified these cases as “hidden suicides.”

### Estimating cumulative incidence of suicide in the study area

We estimated the cumulative incidence of suicide (suicide-specific mortality rate) in the study area over the entire time period using both verbal autopsy-confirmed suicides as well as a combination of verbal autopsy-confirmed suicides and suspected suicides. We standardized each of these outcomes to the average population estimate for the study site over the time period. To obtain a robust population estimate we adjusted the suicide rates for missing data by multiplying the estimate by the inverse of the proportion of deaths in the area without a verbal autopsy. For suicide rates adjusted for missing data we calculate a credibility window as the 95% confidence interval among unadjusted rates multiplied by the inverse of the 95% confidence interval of the proportion of deaths in the area without a verbal autopsy.

### Content analysis

We *a priori* hypothesized the following factors to be associated with suicide: mental illness, dementia, depression, previous reports of self-harm, previous reports of suicide attempts, substance abuse, interpersonal difficulties, financial stress, chronic co-morbidities, domestic violence, legal problems, living alone, a stressful life event, trauma, and experience with the mental health care system. From content analysis of the verbal autopsies, we classified deaths as reporting these factors or not.

### Case-control study

We conducted an unmatched case-control study of suicides using two definition of cases: i) only suicides confirmed by the verbal autopsy, and ii) both suicides confirmed by verbal autopsy and likely suicides identified by content analysis. For controls we selected deaths from external causes (accidents, violence, trauma) as their age profile best reflected the age profile of suicide-caused deaths. We examined the relative association between the presence of hypothesized factors in the verbal autopsies and the two outcomes (confirmed suicides only or suspected and confirmed suicides) using logistic regression. Following the “rule of ten events” [29, 30] we did not examine factors with insufficient sample size, but report their relative infrequency and bivariate association with the outcomes using Fisher’s Exact Test. All analyses were conducted in Stata version 15.1.

## Results

**Fig. 2:**
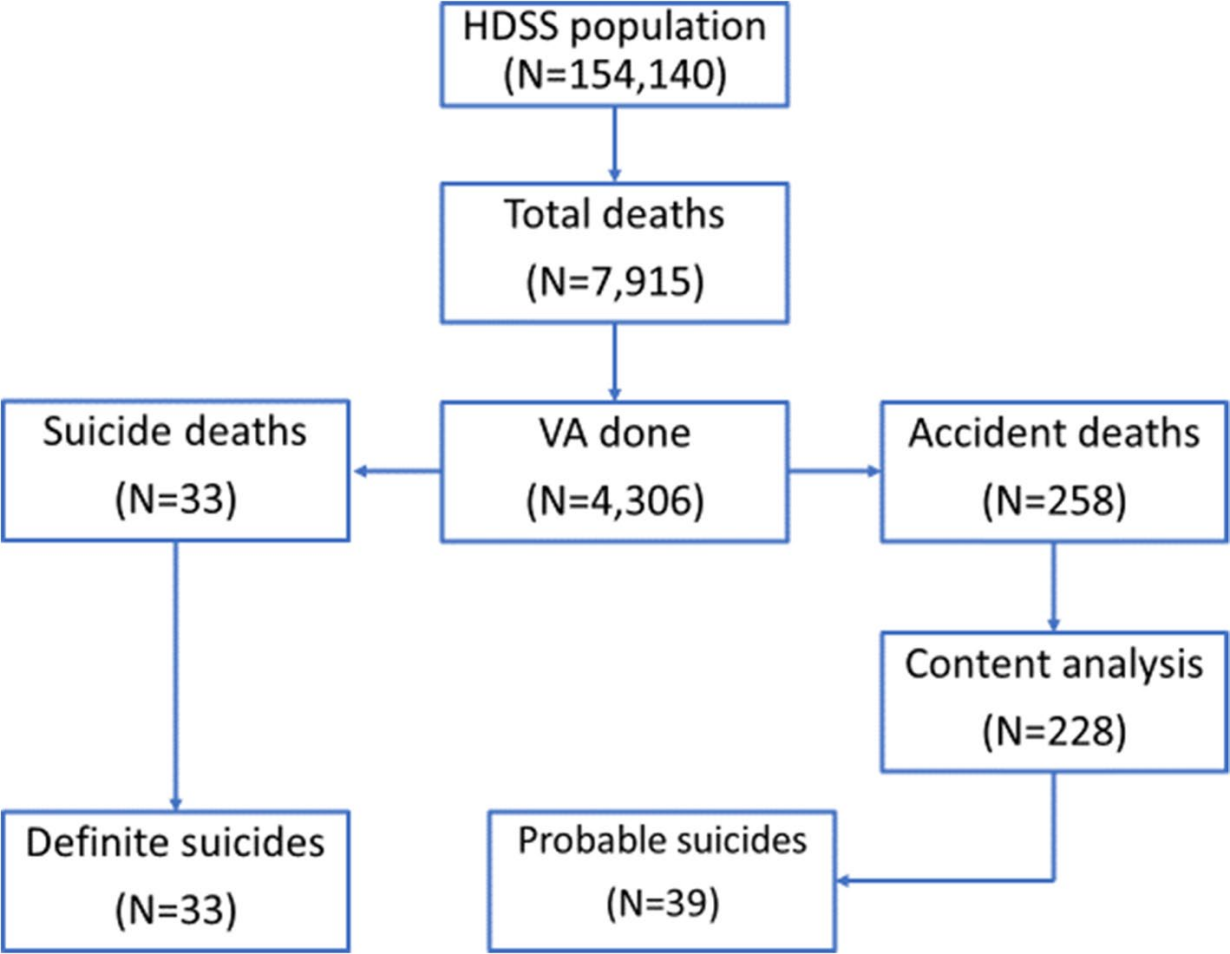
Study flow diagram

From 2011-2017 7,915 deaths were recorded in the HDSS of which 4,306 (54%) had a verbal autopsy. From those deaths that had a verbal autopsy, 291 (7%) were classified as either external cause or suicide. The median age of verbal autopsy-confirmed suicides was 35 years ranging from 17 – 73. Sixty-three accident-caused deaths were excluded because they were younger than 17 years old, resulting in 228 analyzed (Fig. 2).

### Suicide-specific mortality rate

Thirty-three verbal autopsies were confirmed as suicides, giving a cumulative incidence of 3.6 per 100,000 population per year (95% confidence interval [CI] = 2.5 – 5.0 per 100,000). Adjusting for missing data gives an adjusted cumulative incidence of 6.7 per 100,000 population per year (credibility window [CW] = 4.6 – 9.1 per 100,000 population per year). Through content analysis of verbal autopsy narratives, a further 39 deaths were identified as suspected suicides, bringing the estimate of the number of suicides to 72 and the cumulative incidence to 7.9 per 100,000 population per year (95% CI = 6.2 – 9.9 per 100,000 population per year). Adjusting for missing data gives an adjusted cumulative incidence of 14.7 per 100,000 population per year (CW = 11.3 – 18.0 per 100,000 population per year) (Table 1).

**Table 1:** Estimated suicide-specific mortality rate over the time period 2011-2017

Method of estimating number of suicides	Suicide-specific mortality rate per 100,000 population per year (95% confidence interval or credibility window)
**Verbal-autopsy confirmed suicides only**	3.6 (2.5 – 5.0)
**Verbal-autopsy confirmed suicides adjusted for deaths missing verbal autopsy**	6.7 (4.6 – 9.1)
**Verbal-autopsy confirmed and suspected suicides**	7.9 (6.2 – 9.9)
**Verbal-autopsy confirmed and suspected suicides adjusted for deaths missing verbal autopsy**	14.7 (11.3 – 18.0)

### Method of suicide

**Fig. 3:**
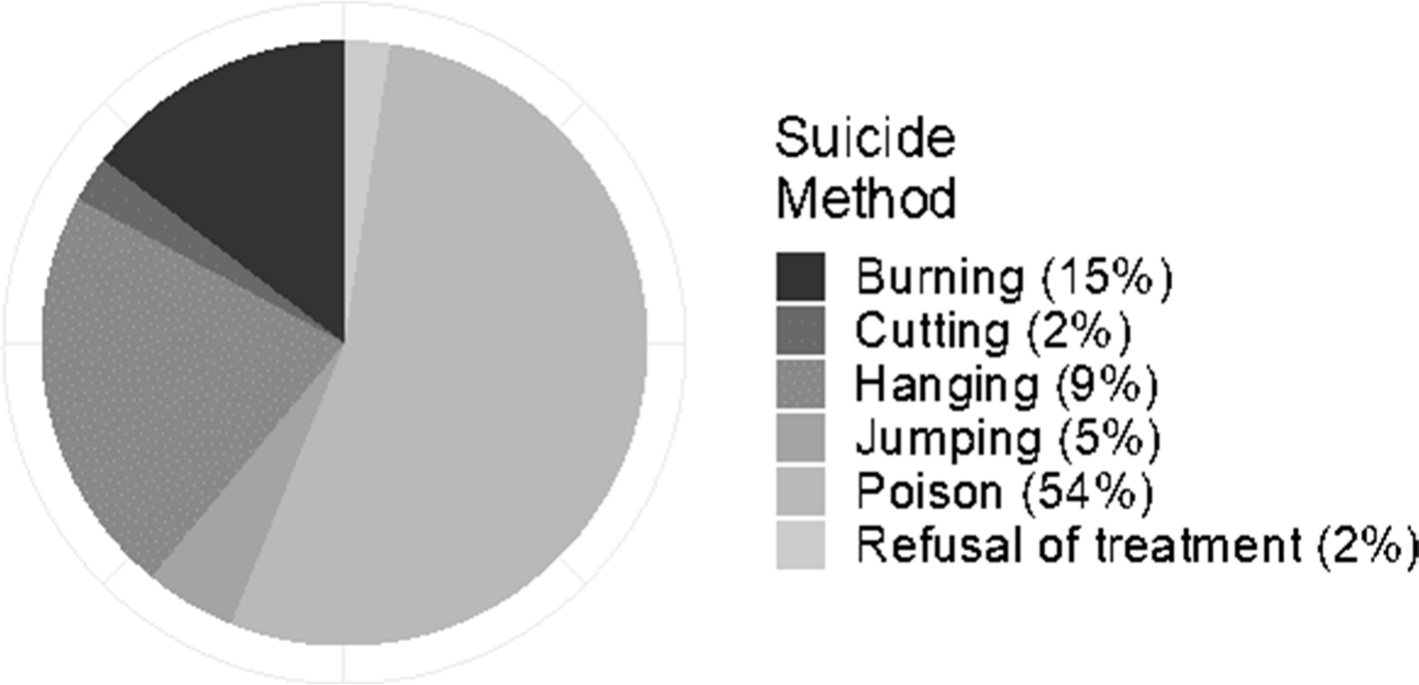
Method of suicide reported in the narrative of 41 verbal autopsies in rural western Kenya

The method of death was noted in the narrative of 29 (88%) verbal autopsy-confirmed suicide cases and 12 (31%) suspected suicide cases from the narrative. The most common reported methods of death were poisoning (54%), burning (15%) and hanging (9%)in a descending order. Among males poisoning and hanging were the most common methods compared to burning and poison in females. Poisoning was mainly from reports of pesticide and organophospate poisoning (Fig. 3).

### Suspected suicides content analysis

From content analysis, numerous factors suggested that the death classified as an external cause by verbal autopsy may have actually been a suicide. These included more factors such as self-harm as well as other factors such as history of mental illness, substance abuse, and stressful life events. Content analysis suggested that 39 (20%) of 195 deaths classified as an accident may have been suicides. (Table 2).

**Table 2:** Distribution of various factors identified during content analysis of verbal autopsy narratives

	Confirmed suicide (%)	Suspected suicide (%)	Not likely suicide (%)
**Male**	28 (85%)	22 (56%)	105 (67%)
**Any Mental illness**	11 (33%)	6 (15%)	3 (2%)
**Dementia**	4 (12%)	5 (13%)	4 (3%)
**Depression**	8 (24%)	0 (0%)	1 (1%)
**Previous self-harm**	0 (0%)	0 (0%)	0 (0%)
**Previous suicide attempts**	1 (3%)	0 (0%)	0 (0%)
**Substance abuse**	9 (27%)	18 (46%)	19 (12%)
**Interpersonal difficulties**	9 (27%)	3 (8%)	6 (4%)
**Financial stress**	0 (0%)	2 (5%)	0 (0%)
**Chronic co-morbidities**	10 (30%)	21 (54%)	36 (23%)
**Domestic violence**	1 (3%)	0 (0%)	3 (2%)
**Legal problems**	0 (0%)	1 (3%)	0 (0%)
**Living alone**	0 (0%)	3 (8%)	1 (1%)
**Stressful life event**	10 (30%)	16 (41%)	7 (4%)
**Trauma**	0 (0%)	1 (3%)	1 (1%)
**Total**	33 (100%)	39 (100%)	156 (100%)

N = 228

### Case-control analysis of factors associated with suicides

Among verbal-autopsy confirmed suicides (excluding those suspected) there was no mention previous instances of self-harm, financial stress, legal problems, living alone, trauma, or experience with the mental health care system as potential factors in the suicide. Being male, younger age, history of mental illness, depression, interpersonal difficulties, and a stressful life event were associated with an increased odds of the death being classified as a suicide on bivariate analysis (Table 3). In adjusted analysis, the odds of dying by suicide was inversely correlated to age and positively with presence of a mental illness and interpersonal difficulties (Table 3).

**Table 3:** Associations between various risk factors mentioned in verbal autopsy narratives and suicide. Comparing verbal autopsy-classified suicides to those classified as an accident

	Bivariate analysis		Adjusted analysis	
**Factor**	**Odds Ratio (95% Confidence Interval) or Fisher’s Exact Test**	**P-value**	**Odds Ratio (95% Confidence Interval)**	*P* **-value**
**Being male**	3.0 (1.1 – 8.1)	0.031	2.7 (0.8 – 8.4)	0.096
**Age (continuous)**	0.97 (0.95 – 0.99)	0.004	0.97 (0.94 – 0.99)	0.012
**Any Mental illness**	10.3 (3.9 – 27.7)	< 0.001	14.2 (4.5 – 45.4)	< 0.001
**Dementia**	FET = 0.100	0.100	Not included	
**Depression**	FET < 0.001	< 0.001	Not included	
**Interpersonal difficulties**	7.8 (2.8 – 21.4)	< 0.001	3.7 (1.0 – 13.1)	0.044
**Chronic co-morbidities**	1.1 (0.5 – 2.4)	0.900	Not included	
**Domestic violence**	FET = 0.467	0.467	Not included	
**Stressful life event**	3.3 (1.4 – 7.7)	0.007	2.9 (0.9 – 9.7)	0.076

N = 228, FET = Fisher’s exact test

Among both verbal-autopsy confirmed suicides and those suspected suicides, male sex, younger age, mental illness, depression, interpersonal difficulties, and a stressful life events were associated with an increased odds of the death being classified as a suicide on bivariate analysis (Table 4). Many of these associations were quite strong, including mental illness, and the limited sample size precluded their inclusion in an adjusted analysis. In adjusted analysis, the odds of dying through suicide increased with chronic comorbidities and stressful life events (Table 4).

**Table 4:** Various risk factors mentioned in verbal autopsy narratives and suicide. Comparing both verbal autopsy-classified suicides and suspected suicides identified by content analysis to those classified as an accident

	Bivariate analysis		Adjusted analysis	
**Factor**	**Odds Ratio (95% Confidence Interval) or Fisher’s Exact Test**	**P-value**	**Odds Ratio (95% Confidence Interval)**	**P** **-value**
**Being male**	1.1 (0.6 – 2.0)	0.748	Not included	
**Age (continuous)**	1.00 (0.99 – 1.01)	0.703	Not included	
**Mental illness**	FET < 0.001	< 0.001	Not included	
**Depression**	FET < 0.001	< 0.001	Not included	
**Dementia**	FET = 0.005	0.005	Not included	
**Interpersonal difficulties**	FET = 0.002	0.002	Not included	
**Financial stress**	FET = 0.099	0.099	Not included	
**Chronic co-morbidities**	2.5 (1.4 – 4.6)	0.002	2.0 (1.0 – 3.8)	0.043
**Domestic violence**	FET = 1.00	1.00	Not included	
**Living alone**	FET = 0.094	0.094	Not included	
**Stressful life event**	12.0 (4.9 – 29.5)	< 0.001	10.7 (4.3 – 26.5)	< 0.001

N = 228

## Discussion

This study sought to comprehensively investigate the burden and to characterize suicide in Western Kenya using content analysis of verbal autopsies. The annual suicide–specific mortality rate for confirmed cases in the study population was 3.6 per 100,000, similar to the rate on coastal Kenya (4.2/100,000) [31]. Our finding is relatively lower than the estimated WHO national rate (6.1 per 100,00) and regional age-standardized rate of 11.2 per 100,00 0[2] . There is a general consensus that suicide statistics are largely an underestimate, especially in LMICs where consistent and systematic systems of reporting suicide cases are lacking [32, 33]. Previously, other exemplary scholars have improved the accuracy of suicide completion estimates by adjusting for unreported deaths and projecting these estimates to corresponding populations [34]. Under reporting and misclassification of suicide is evidenced in our study by the finding that, after combining confirmed and suspected cases and adjusting for missing data, we found a fourfold (14.67 per 100,000) increase compared to the confirmed suicide mortality rate of 3.6 per 100,000 populations. This adjusted rate is closer to regional rates estimation for 2019 of 11.2 deaths per 100,000 populatio n[2]. Additionally, the 15% reduction in open history form completion in cases that were classified as suicide, external causes, or the reporter answered yes to “Do you think the deceased committed suicide?” may further lead to underreporting. Social, cultural and legal factors contribute significantly to under reporting and misclassification of suicide cases in Kenya [4], as suicide is culturally highly stigmatized and suicidal attempts remain a punishable offence in Kenya [10, 35, 36]. To avoid any repercussion, close relatives to the deceased may be averse to overtly disclosing suicide as cause of death during verbal autopsies.

The finding that poisoning is the most common suicide method in our study population corroborates what has been reported in many studies from LMICs including Africa [37] with no gender bias. Suicide by poisoning is reported to be especially common in the rural areas of LMICs similar to our study setting, and this has been attributed to the availability of toxic agricultural pesticides [38]. However, a study by Bitta et al on the coast of Kenya found hanging to be the most common method of suicide [31]. This variation may be due to cultural differences between the two communities. In contrast, to high income countries, where firearm use is the second commonest method of suicide used after hanging [39], our study found no firearm use report. Generally, firearm use as a common method of suicide is varied in the sub- Saharan region, largely based on availabilit y[39]. Reports of firearm use as a suicide method have been increasing in Kenya, these are often limited to the police and other law enforcement officers, due to the ease of access [40]. Our study region being a rural and resource poor setting, means many lack access to firearms. Curiously, our study found 15% of the study population had used burning or self-immolation as a suicide method. Burning is regarded as a violent and extreme suicide method that rarely gets mentioned as it is considered an uncommon means of suicide globall y[41]. Despite this, other studies have found surprisingly higher occurrence of suicide by self-immolation for example a study in South Africa reported a 9.1% prevalenc e[42]. Aside from cultural factors, suicide by burning has been previously associated with presence of adjustment disorders, psychoses, or substance use disorder s[43].

History of mental illness was the strongest risk factor associated with suicide with others such as stressful life events and interpersonal difficulties also contributing. This finding of mental illness being a major risk factor has also been reported by other studies [44] with the commonest illnesses being depression, substance use and psychotic disorders [45]. Over 90% of persons who die by suicide have a diagnosable mental disorder [46]. The treatment gap for mental illness is high in sub-Saharan Africa thereby contributing further to higher suicide risk in the region [47]. The Kombewa region where the HDSS is located, lacks a dedicated mental health unit, hence, likely many cases of mental illness may go unidentified and untreated. This could further explain the high suicide rates found in the area following content analysis. However, contrary to our findings, other studies have raised questions on how substantial the role of mental illness is on suicide especially in some LMICs where considerable number of suicides have been found to occur impulsively in people without any diagnosable mental illness [48]. Suggesting some variability in specific population characteristics and the need for multipronged suicide prevention interventions addressing various risk factors [49]. High levels of perceived stress have been shown to be a trigger of suicide especially in vulnerable individuals [50]. Although our study was not able to analyze for individual specific stressors, the Jenkins et al study on non-fatal suicidal behavior in the same HDSS setting similarly found that the number of stressful life events was a strong predictor of suicidal behavior [51]. A thwarted sense of belongingness posited in the interpersonal theory of suicide increases suicide risk [52, 53]. Correspondingly, in the adjusted analysis reports of interpersonal difficulties increased suicide risk three-fold. Being male was associated with increased odds of a death being classified as suicide. This sex difference is comparable to many studies that attribute this phenomenon to lethality of means as well as increased access to these means for males compared to females [54, 55]. While poisoning was high for both groups, we found males were more likely to have died from hanging compared to females. In addition to the lethality of means, difference in health seeking behavior and engagement in maladaptive coping mechanism like substance abuse increase the risk of suicide in males [56–58]. Suicide risk decreased with increasing age in our study. Age associated trends in suicide rates are varied even in Africa [4, 59], however a general increase in suicide rates among young people and decrease in the elderly has been noted over the past 50 years [60]. The WHO 2016 report indicated a peak age in suicide rates to be between 20-25 years, a second slight peak at about 50 years followed by a decrease in rates with increasing age for LMICs [61]. In the majority of studies in sub-Saharan Africa there is a consistently high rate of suicide among young adults (15-30 years) [4]. Barriers to accessing health care compounded by the stigma and fear associated with seeking for help for suicide attempts and mental disorders may be key contributors to this trend. It is noteworthy that mental disorders are strongly linked to suicidal behavior and a greater proportion of these disorders have their onset by the mid-20s [62].

Factors associated with suicide remained similar even after combining confirmed and suspected cases. This further underscore the importance of content analysis in estimating suicide rates in verbal autopsy studies to avoid under reporting of suicide.

There are some limitations in these analyses, primarily the incomplete verbal autopsy data for the HDSS. We suspect the verbal autopsy data to be missing at random, or at least due to convenience of the HDSS team rather than to be associated with the type of death reported. We do not expect any bias to be present, given the relatively stable rate of suicides reported despite the variable rate of verbal autopsies conducted. Secondly, there may be some overlap between “any mental illness” and the specific mental illnesses identified by the interviewers. The threshold for illnesses identified by the interviewers at autopsy interview may be different from those that would have operated had the subjects been given a gold standard interview before their death, with possible over identification as well as under identification. Further, there may not have been sufficient information for all verbal autopsies, hence it is possible to underestimate the effect size of some risk factors if not all cases were filtered from the comparison group. Our small sample size may be limiting for the advanced analysis of identifying factors associated with suicide, however suicide being a rare occurrence in general population data, coupled with scarcity of similar data in LMIC informed our decision to further undertake association analysis. Lastly, suicide cases reported in our estimates were not confirmed by postmortem reports, we instead relied on verbal autopsy reports from the relevant households.

## Conclusion

Suicide rates in Western Kenya mirror the epidemiology found in other LMICs. After combining confirmed and suspected deaths from suicide, we found a high suicide rate in this population. There is need for better comprehensive and systematic reporting of suicide in the region coupled with mental health promotion and prevention of mental illnesses given the significant association of mental illness and suicide in our study. Content analysis provides insight into the nuances of mental illness and suicidal behaviors in this setting which should be used to tailor suicide prevention programs. Additionally, the village reporter model that the HDSS employs provides an infrastructure to expand access to mental health care through task shifting. In this region, community health workers and village reporters are a readily available and trusted resource that provide a variety of functions including outreach, counseling, health promotion, and referral. Suicide prevention strategies in the area should also empower CHWs and village reporters to screen for mental health conditions as a means to increase awareness and access to mental health care services.

## Data Availability

The datasets used and analyzed during the current study are available from corresponding author on reasonable request.

## Abbreviations

CHWs: Community Health Workers
HDSS: Health and Demographic Surveillance System
HIV: Human Immunodeficiency Virus
ICD-10: International Classification of Diseases version 10
KEMRI: Kenya Medical Research Institute
LMIC: Low and Middle Income Country
WHO: World Health Organization
WRAIR: Walter Reed Army Institute of Research
USMRD-K: United States Medical Research Directorate-Kenya
VA: Verbal Autopsy
